# Eco-Friendly Synthesis of Silver Nanoparticles Using *Lespedeza capitata* Extract: Antioxidant and Anti-Inflammatory Properties in Zebrafish (*Danio rerio*)

**DOI:** 10.3390/ijms262110693

**Published:** 2025-11-03

**Authors:** Roxana Delia Chitiala, Ionut Iulian Lungu, Andreea-Maria Mitran, Ioana Mita-Baciu, Ion Brinza, Cornelia Mircea, Anisoara Nistor, Monica Hancianu, Radu Iliescu, Lucian Hritcu, Oana Cioanca

**Affiliations:** 1 Grigore T. Popa University of Medicine and Pharmacy Iasi, 16 Universitatii Street, 700115 Iasi, Romaniaoana.cioanca@umfiasi.ro (O.C.); 2 Faculty of Sciences, “Lucian Blaga” University of Sibiu, 7–9 Ion Ratiu Street, 550024 Sibiu, Romania; 3 Human Vanelli, VANELLI SRL, Iasi 707280, Romania; 4 Faculty of Biology, Alexandru Ioan Cuza University of Iasi, Bd. Carol I, No. 11, 700506 Iasi, Romania

**Keywords:** silver nanoparticles, zebrafish, antioxidant, green synthesis

## Abstract

Silver nanoparticles (AgNPs) were synthesized using a modified literature method involving aqueous AgNO_3_ (3 mM) and plant extract (LCE) at a constant ratio, under alkaline conditions and controlled temperature. The nanoparticles were characterized by UV-Vis spectroscopy, dynamic light scattering (DLS), zeta potential analysis and scanning transmission electron microscopy (STEM). The UV-Vis spectra displayed a broad absorption band around 450 nm, indicative of polydispersity, while DLS revealed a hydrodynamic diameter of 90.3 nm with a polydispersity index of 0.3366. Zeta potential values suggested reduced electrostatic stability compared with previously reported plant-derived AgNPs, although STEM images confirmed predominantly spherical, well-dispersed nanoparticles with sizes between 15 and 20 nm. Functional assays in zebrafish demonstrated the biological relevance of AgNPs. In scopolamine-induced models of cognitive and behavioral deficits, AgNPs treatment significantly improved memory and locomotor activity, as assessed by the Y-Maze, Novel Tank Diving Test and Novel Object Recognition Test.

## 1. Introduction

*Lespedeza capitata* Michx., commonly known as roundhead bushclover, is a perennial herbaceous species of the Fabaceae family, native to the prairies and open woodlands of North America. It is well adapted to dry, sandy and nutrient-poor soils, contributing significantly to soil fertility through its nitrogen-fixing symbiosis with rhizobia. Beyond its ecological role as a forage and soil-improving species, *L. capitata* has been reported in ethnomedicine for its diuretic, hepatoprotective and anti-inflammatory effects. Phytochemical analyses have revealed that species of the *Lespedeza* genus, including *L. capitata*, are rich in polyphenolic compounds such as flavonoids, isoflavones, phenolic acids and tannins, alongside saponins and alkaloids that may contribute to diverse pharmacological activities. These metabolites are associated with strong antioxidant potential, radical scavenging properties and the modulation of inflammatory mediators, thereby suggesting a protective role against oxidative stress-related diseases [1,2,3,4].

Silver nanoparticles (AgNPs) have been thoroughly studied in recent years due to their unique physicochemical features, which result from their nanoscale size and surface-related effects. Their elevated surface-area-to-volume ratio, adjustable morphology and significant surface plasmon resonance effects provide improved catalytic activity, electrical conductivity and optical responsiveness, while their established antimicrobial, antiviral and antifungal properties render them ideal for biomedical, pharmaceutical and environmental applications [5,6,7].

Traditional synthesis processes such as chemical reduction necessitate toxic reagents and considerable energy consumption and produce environmentally lasting byproducts, constraining their sustainability and biocompatibility. In contrast, plant-mediated synthesis has arisen as a viable green alternative, utilizing the natural prevalence of phytochemicals including phenolics, flavonoids, terpenoids and alkaloids, which function concurrently as reducing and stabilizing agents. This biogenic method not only removes the necessity for hazardous chemicals but also improves nanoparticle stability and biocompatibility by providing a biological profile from plant metabolites [8,9].

Plant extract-mediated AgNPs constitute an eco-friendly and functionally adaptable platform with considerable potential in nanomedicine, diagnostics and therapeutic applications.

There is increasing evidence that oxidative stress and chronic inflammation are strongly implicated in the pathogenesis of neurodegenerative disorders, including mild cognitive impairment (MCI), which can progress to dementia. These processes may be modulated not only by lifestyle interventions but also through the use of bioactive compounds with antioxidant and anti-inflammatory activity. Plant-derived metabolites are particularly attractive in this regard, as they provide a rich source of natural agents with therapeutic potential. However, their poor bioavailability often limits clinical translation, thereby justifying innovative strategies such as nanoformulation to enhance stability and biological efficacy [10,11,12].

The main objective of this study was to attempt to increase the antioxidant and biomedical properties of *Lespedeza capitata* extract by synthesizing silver nanoparticles with an acceptable size and morphology as well as increased effects in scopolamine-treated zebrafish.

## 2. Results and Discussions

### 2.1. Physico-Chemical and Morphological Characteristics

The UV-Vis absorption spectra of the nanoparticle sample show unique optical behaviors which indicate changes in their structural and electronic properties.

**Figure 1:**
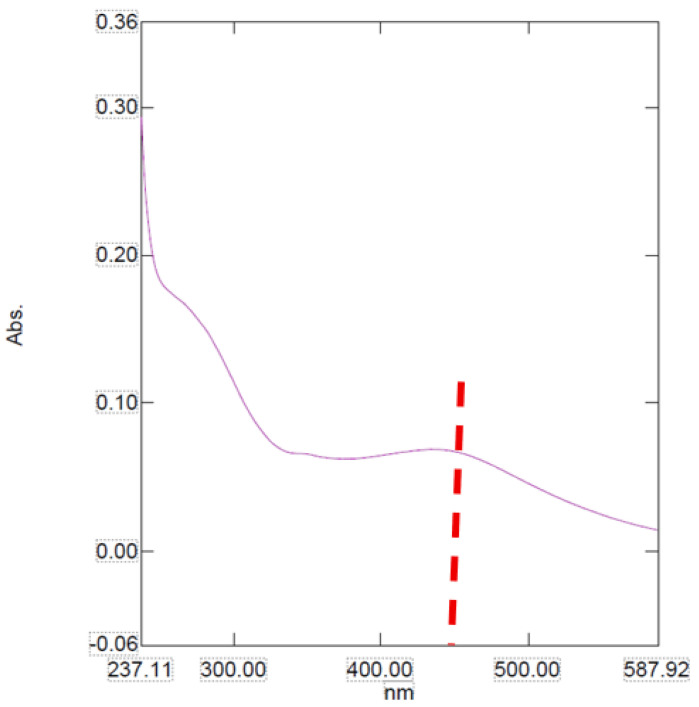
The UV-Vis spectra for AgNPs. **Note:** The red line represents the NP peak.

The absorption spectra for the sample presents a broader and less pronounced profile with a maximum absorbance around 450 nm (Figure 1). This extensive and uniform absorption pattern may be due to a more amorphous structure or a high degree of polydispersity in the nanoparticle size population [13].

**Figure 2:**
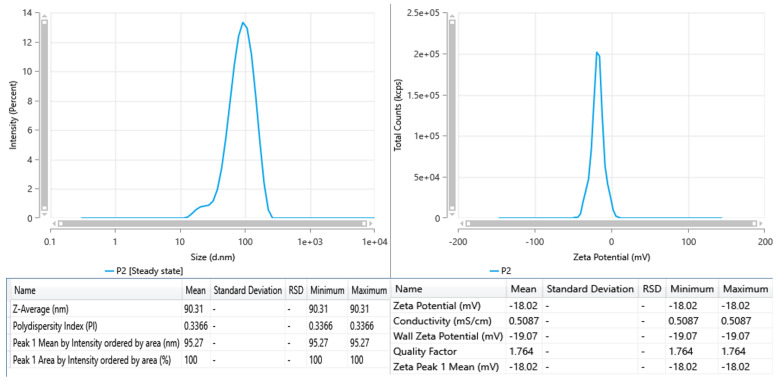
DLS and zeta potential for AgNPs.

The DLS tests for the sample indicate a singular distinct distribution peak centered around 95.3 nm, with a Z-average hydrodynamic diameter of 90.3 nm (Figure 2). The PDI of 0.3366 signifies a somewhat polydisperse system; however, it remains within an acceptable range for colloidal suspensions. The lack of secondary peaks shows that the particle distribution is reasonably homogeneous and devoid of substantial aggregates or larger particles.

The sample is distinguished by slightly bigger and mostly monomodal particles; nonetheless, its reduced zeta potential indicates diminished electrostatic stability.

For comparison, AgNPs synthesized using flaxseed extract, with a zeta potential of −44 mV, exhibit very strong electrostatic stabilization [14]. Literature findings also indicate that zeta potentials over 20 mV typically ensure good colloidal stability; their nanoparticle solutions can be deemed as stable nanosuspensions for biomedical testing [15].

**Figure 3:**
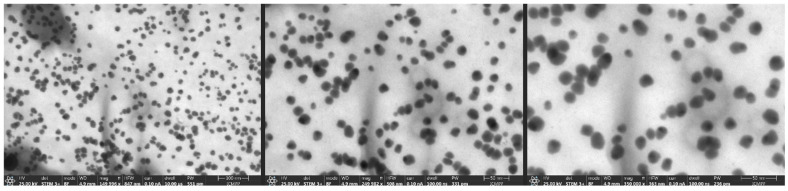
STEM images for the nanoparticle sample.

The STEM images for the sample (Figure 3) exhibit a significantly more homogeneous particle morphology and distribution. The nanoparticles primarily present as spherical and exhibit good dispersion, showing minor evidence of agglomeration. The size distribution in the images confirms the DLS results, which suggested a mean hydrodynamic diameter of around 90.3 nm and a PDI of 0.3366. The STEM images present spherical nanoparticles with average sizes from 15 to 20 nm; each individual nanoparticle is separate and perfectly dispersed in liquid.

STEM imaging offers visual validation of the trends and predictions suggested by the DLS and zeta potential investigations. The nanoparticles have enhanced morphological uniformity and superior dispersion, indicated more consistent particle formation or enhanced colloidal stability under the used microscope conditions.

The hydrodynamic diameter obtained from DLS analysis (90.3 nm) was considerably larger than the particle size estimated from STEM images (15–20 nm). This difference can be attributed to the distinct principles of the two techniques. DLS measured the apparent diameter of nanoparticles in colloidal suspension, which includes not only the metallic core but also the surrounding solvation layer and any biomolecules adsorbed on the particle surface. In the case of green-synthesized AgNPs, phytochemicals from the plant extract (such as phenolic, flavonoids and proteins) act as capping and stabilizing agents, forming an organic corona around the nanoparticle. STEM provides a measure of the metallic core, excluding the surrounding organic components.

### 2.2. Antioxidant Activity

#### 2.2.1. Ferrous Ion Chelation

**Figure 4:**
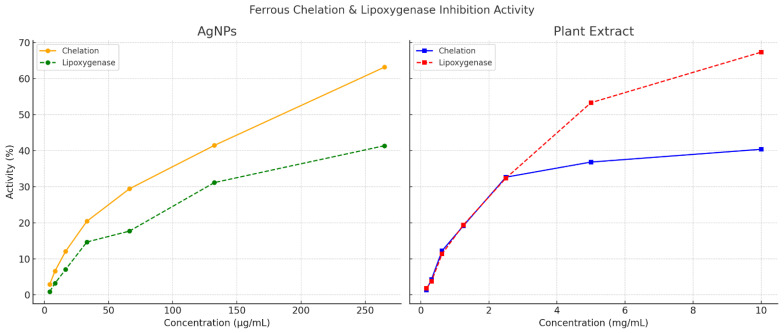
Ferrous ion chelation and lipoxygenase inhibition for the plant extract and AgNPs.

Phytochemical screening of the *Lespedeza capitata* extract revealed the presence of both hydrophilic and lipophilic compounds, with a predominance of lipophilic metabolites. Since such compounds typically display reduced oral bioavailability, nanoformulation offers a significant advantage by improving solubility, absorption and cellular uptake [1,16]. In the present study, AgNPs synthesized using the plant extract displayed markedly enhanced biological effects compared with the crude extract. Specifically, antioxidant assays (Table 1, Table 2 and Table 3) demonstrated that the nanoparticles exhibited at least a ten-fold stronger activity in both ferrous ion chelation and lipoxygenase inhibition assays (Figure 4).

#### 2.2.2. Hydroxyl Ion Scavenging

The antioxidant effects of the AgNPs can be explained through two possible mechanisms that stem from the nanosinzing of the particles but also from the synergism that the metallic cores have with the corona made from phytochemicals like phenolics and organic acids, efficiently neutralizing oxidants. Ferrous ions are central mediators in Fenton-type reactions, generating reactive oxygen species that drive oxidative stress; the ability of nanoparticles to efficiently chelate these ions thus highlights their protective potential.

#### 2.2.3. Lipoxygenase Inhibition Activity

Similarly, lipoxygenase is a key enzyme in inflammatory processes, and its overexpression has been linked to cancer, neurodegenerative disorders and other pathologies [17].

Interestingly, the crude extract alone exhibited comparable potency at lower concentrations in both antioxidant tests, suggesting the inherent activity of its phytochemical constituents. However, at higher concentrations, the nanoparticles clearly outperformed the extract, underlining the added value of the nanoformulation. These findings are consistent with previous studies reporting the enhanced biological activity of plant-derived compounds when incorporated into nanostructured systems [18,19,20,21,22]. Future studies should focus on evaluating additional inflammatory and oxidative stress markers in order to provide a more comprehensive understanding of the therapeutic potential of these biogenic nanoparticles.

### 2.3. Animal Tests

#### 2.3.1. Y-Maze Test

**Figure 5:**
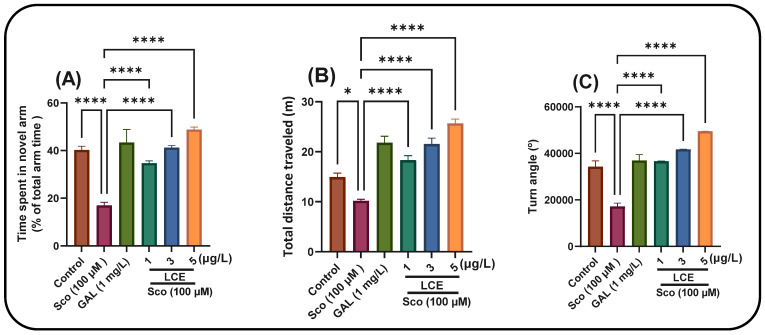
Assessment of response to novelty in Sco (100 µM)-treated zebrafish subjected to LCE treatment at doses of 1, 3 and 5 μg/L in the Y-maze test. (**A**) Time spent in the novel arm (% of total arm time); (**B**) Total distance traveled (m); (**C**) Turn angle (°). Data are presented as means ± S.E.M. (*n* = 10 animals per group). * *p* < 0.0 and **** *p* < 0.00001 (Tukey’s post hoc analyses). Galantamine (GAL, 1 mg/L) was used as a reference positive drug.

**Figure 6:**
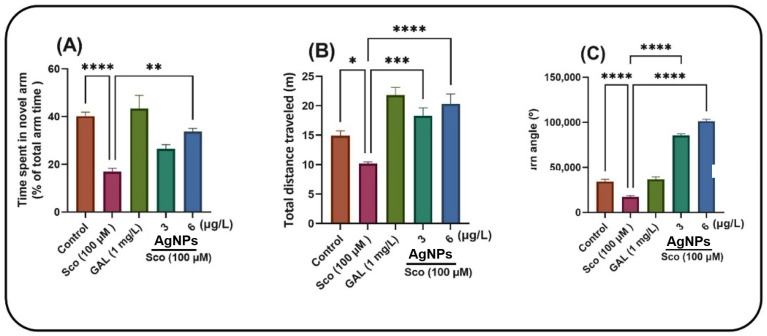
Assessment of the response to novelty in Sco (100 µM)-treated zebrafish subjected to AgNPs treatment at doses of 3 and 6 μg/L in the Y-maze test. (**A**) Time spent in the novel arm (% of total arm time), indexing of spatial memory; (**B**) Total distance traveled (m), indexing locomotion; (**C**) Turn angle (°), indexing exploratory strategy. Data are presented as means ± S.E.M. (*n* = 10 animals per group). * *p* < 0.01, ** *p* < 0.001, *** *p* < 0.0001 and **** *p* < 0.00001 (Tukey’s post hoc analyses). Galantamine (GAL, 1 mg/L) was used as a reference positive drug.

To evaluate the impact of LCE on the novelty response, zebrafish were examined using a Y-maze to measure the duration spent in the novel arm. The one-way ANOVA indicated that the treatment significantly influenced the time allocated to the novel arm [F (5, 54) = 19.77] (*p* < 0.0001) (Figure 5A), total distance traversed [F (5, 54) = 34.23] (*p* < 0.0001) (Figure 5B) and turn angle [F (5, 54) = 46.11] (*p* < 0.0001) (Figure 5C). Zebrafish subjected to the Sco treatment exhibited significantly reduced time spent in the novel arm compared to the control group (*p* < 0.00001). Compared to the zebrafish treated solely with Sco, LCE therapy markedly mitigated the amnesic impact, with *p* values of 0.00001 for concentrations of 1, 3 and 5 μg/L, respectively. Zebrafish exposed to Sco demonstrated reduced locomotor activity, evidenced by a substantial decrease in total distance traveled (*p* < 0.01) (Figure 6B) and turn angle (*p* < 0.00001) (Figure 6C) relative to the control group. Furthermore, LCE treatment in Sco-induced zebrafish enhanced locomotion in the Y-maze, evidenced by a significant increase in total distance traveled (*p* < 0.00001 for 1, 3 and 5 μg/L) and turn angle (*p* < 0.00001 for 1, 3 and 5 μg/L). The observed behavioral recovery may be attributed to the presence of polyphenolic compounds such as kaempferitrin (lespedin), quercetin and rosmarinic acid, previously identified in *L. capitata* extracts [4]. Kaempferitrin exhibits antidepressant- and anxiolytic-like activity mediated via 5-HT_1_A receptor modulation [23], while quercetin and rosmarinic acid have been reported to enhance cholinergic neurotransmission and reduce oxidative stress in the zebrafish brain [24]. Together, these mechanisms may explain the combined cognitive and locomotor improvement observed following LCE exposure.

Furthermore, green-synthesized AgNPs produced using LCE (see Figure 6) displayed similar behavioral trends, reinforcing the hypothesis that nanoformulation enhances the neuroprotective efficacy of phytochemicals by improving bioavailability and stability [25]. Overall, these findings suggest that LCE and its AgNPs derivatives exert potent neuroprotective effects through synergistic antioxidant, anti-inflammatory and cholinergic modulatory actions, aligning with previously reported data on biogenic nanoparticles and medicinal plant extracts in zebrafish models of cognitive impairment [26]

**Figure 7:**
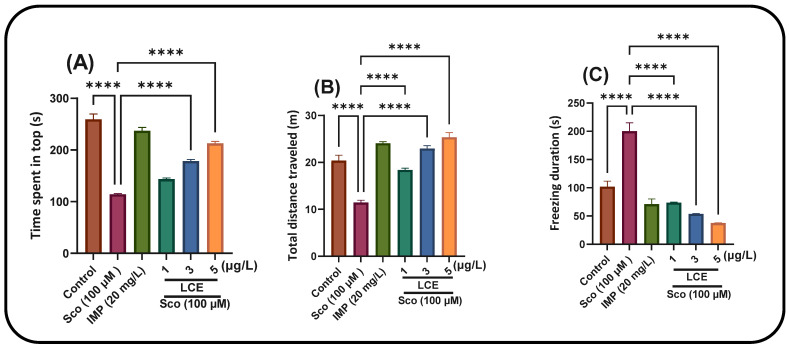
The effects of LCE (1, 3 and 5 μg/L) administration in scopolamine (Sco, 100 µM)-treated zebrafish on anxiety behavior evaluated within the novel tank diving test (NTT). Anxiety response: time spent in the top (s) and freezing duration (s) in different groups. Locomotion: total distance traveled (m) in different groups. Values are means ± S.E.M. (*n* = 10). For Tukey’s post hoc analyses, **** *p* < 0.00001.

Zebrafish were assessed using the Y-maze to examine their responsiveness to novelty after treatment with AgNPs. The one-way ANOVA indicated that the treatment exerted a significant overall effect on the duration spent in the novel arm [F (4, 45) = 14.51, *p* < 0.0001] (Figure 7A), total distance traversed [F (4, 45) = 15.54, *p* < 0.0001] (Figure 7B) and turn angle [F (4, 45) = 307.09, *p* < 0.0001] (Figure 7C). Zebrafish subjected to the Sco treatment exhibited significantly reduced time in the novel arm compared to the control group (*p* < 0.00001). Compared to the zebrafish treated just with Sco, the administration of AgNPs markedly mitigated this amnesic effect, yielding a *p*-value of 0.001 for 6 μg/L. Zebrafish exposed to Sco demonstrated reduced locomotor activity, evidenced by a substantial decrease in total distance traveled (*p* < 0.01) (Figure 7B) and turn angle (*p* < 0.00001) (Figure 7C) relative to the control group. Furthermore, AgNPs administration in Sco-induced zebrafish enhanced locomotion in the Y-maze by increasing the total distance traveled (*p* < 0.00001 for 3 and 6 μg/L) and the turn angle (*p* < 0.00001 for 3 and 6 μg/L).

#### 2.3.2. Novel Tank Diving Test (NTT)

**Figure 8:**
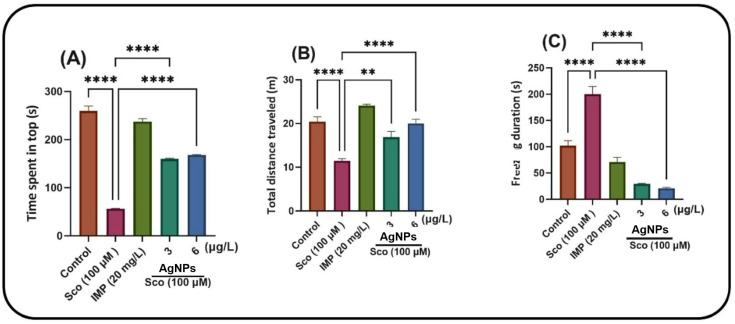
The effects of AgNPs (3 and 6 μg/L) administration in scopolamine (Sco, 100 µM)-treated zebrafish on anxiety behavior were evaluated within the novel tank diving test (NTT). Anxiety response and hypoactivity: time spent in the top (s) and freezing duration (s) in different groups. Locomotion: total distance traveled (m) in different groups. Values are means ± S.E.M. (*n* = 10). For Tukey’s post hoc analyses, ** *p* < 0.001 and **** *p* < 0.00001.

The NTT evaluates the anxiety response triggered by novelty. In the NTT test, one-way ANOVA demonstrated a significant effect of the treatment on the time spent in the top zone of the tank (F (5, 54) = 107.4, *p* < 0.0001) (Figure 8A), total distance traveled (F (5, 54) = 50.25, *p* < 0.0001) (Figure 8B) and freezing duration (F (5, 54) = 50.79, *p* < 0.0001) (Figure 8C). Sco treatment markedly reduced the duration at the upper zone of the tank (*p* < 0.00001) and augmented freezing behavior (*p* < 0.00001) relative to the control group. The reduction in total distance traveled (*p* < 0.00001) and Sco therapy resulted in a hypolocomotor effect relative to the control group. The anxiolytic-like effect of LCE treatment (1, 3, and 5 μg/L) was observed by an increase in the duration spent in the upper zone of the tank (*p* < 0.00001) and a reduction in freezing duration (*p* < 0.00001) relative to the Sco-alone treated animals. Furthermore, LCE administration (1, 3 and 5 μg/L) mitigates the hypolocomotor effects of Sco, as seen by the increased total distance traveled (*p* < 0.00001) in comparison to fish treated just with Sco. Imipramine (IMP, 20 mg/L), a positive reference compound, markedly enhanced the duration spent in the upper zone of the tank and reduced freezing time, indicating a diminution of the anxiogenic reaction. Nonetheless, the anxiolytic reaction, concomitant with the enhancement of locomotor activity, was evidenced by a substantial increase in the total distance traversed by the zebrafish in the tank when Sco-alone treated zebrafish were subjected to LCE administration.

Moreover, lespedin (recognized as kaempferitrin), rosmarinic acid and quercetin were among the identified compounds in the investigated extract [1,4]. Such compounds have previosly demonstrated an impact on CNS and their mechanism of action is correlated to anxyolitic and atidepressant-like effects. Murine models have indicated that lespedin has specific antidepressant activity, more effective than fluoxetine or imipramine, known serotonin reuptake inhibitors. Such results have postulated that some of the effectivness of kaempferitrin may be explained by the possible activation of serotonin receptors subtypes (5-HT_1A_) [23].

In the AgNPs treatment, the NTT test indicated that one-way ANOVA demonstrated a significant impact of the treatment on the duration spent in the upper zone of the tank [F (4, 45) = 300.1, *p* < 0.0001] (Figure 8A), on the total distance traversed [F (4, 45) = 25.37, *p* < 0.0001] (Figure 8B) and on the duration of freezing [F (4, 45) = 64.69, *p* < 0.0001] (Figure 8C). Sco administration markedly reduced the duration in the upper zone of the tank (*p* < 0.00001) (Figure 8A) and enhanced freezing behavior (*p* < 0.00001) (Figure 8C) relative to the control group. Sco therapy resulted in a hypolocomotor effect by reducing the total distance walked (*p* < 0.00001) (Figure 8B) in comparison to the control group. The anxiolytic-like effect of AgNPs (3 and 6 μg/L) treatment was observed through an increased duration in the upper zone of the tank (Figure 8A) (*p* < 0.00001) and a reduction in freezing duration (Figure 8C) (*p* < 0.00001) relative to the Sco-alone treated subjects.

Furthermore, the administration of AgNPs (3 and 6 μg/L) mitigates the hypolocomotor effects of Sco, as seen by the increased total distance traveled (Figure 8B) (*p* < 0.001 for 3 μg/L and *p* < 0.00001 for 6 μg/L) in comparison to fish treated solely with Sco. Imipramine (IMP, 20 mg/L), a positive reference compound, markedly enhanced the duration spent in the upper zone of the tank and reduced freezing time, indicating a reduction in the anxiogenic reaction. Nonetheless, the anxiolytic response, concomitant with the enhancement of locomotor activity, was evidenced by a substantial increase in the total distance traversed by the zebrafish in the tank when Sco-alone-treated zebrafish were subjected to AgNPs administration.

#### 2.3.3. Novel Object Recognition Test (NOR)

**Figure 9:**
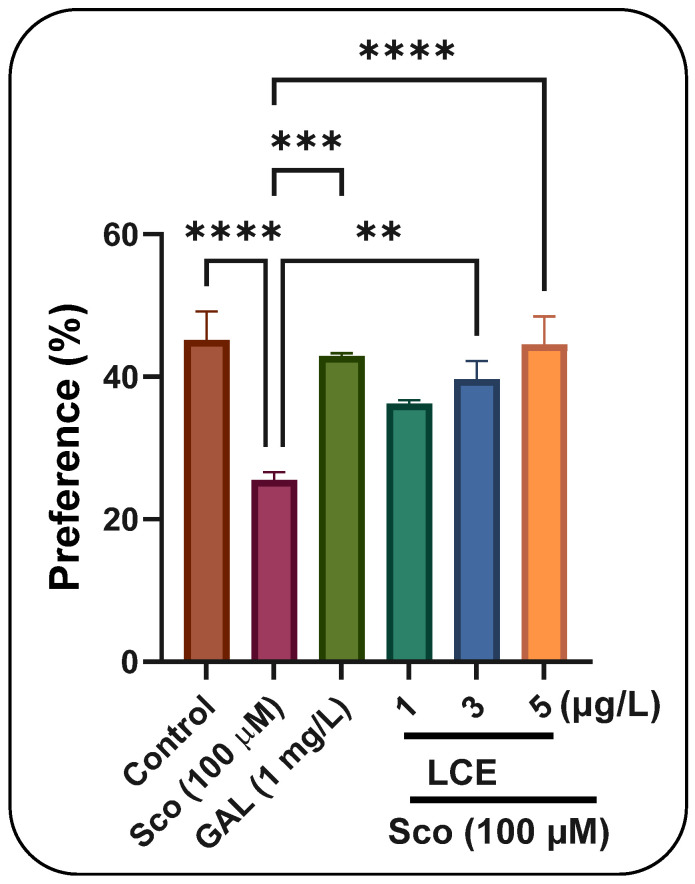
The effects of LCE (1, 3 and 5 μg/L) administration in scopolamine (Sco, 100 µM)-treated zebrafish on the preference percentage evaluated within the novel object recognition test (NOR). Values are means ± S.E.M. (*n* = 10). For Tukey’s post hoc analyses, **** *p* < 0.00001, *** *p* < 0.0001 and ** *p* < 0.001.

**Figure 10:**
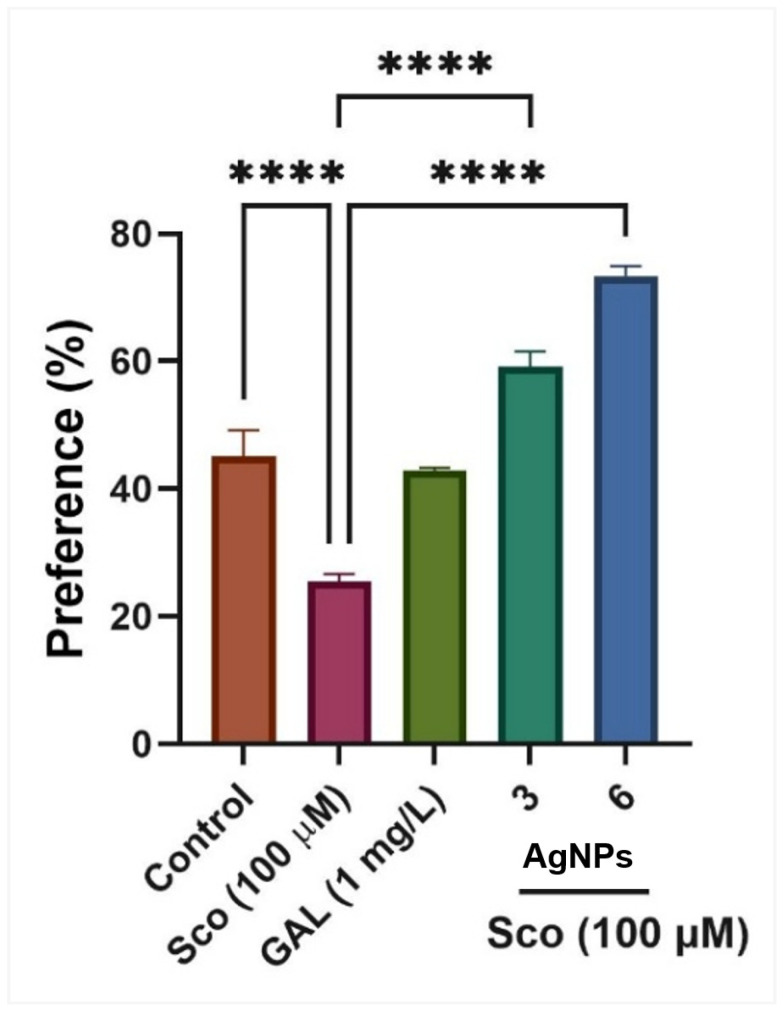
The effects of AgNPs (3 and 6 μg/L) administration in scopolamine (Sco, 100 µg/M)-treated zebrafish on the preference percentage evaluated within the novel object recognition test (NOR). Values are means ± S.E.M. (*n* = 10). For Tukey’s post hoc analyses, **** *p* < 0.00001.

In the NOR test, one-way ANOVA indicated a significant effect of treatment on preference percentages (F (5, 54) = 8.24, *p* < 0.0001) (Figure 9). Zebrafish administered Sco exhibited a dramatic reduction in preference percentages (*p* < 0.0001) relative to the control group, but the administration of LCE, particularly at dosages of 3 μg/L and 5 μg/L, enhanced preferences for the novel item (N), indicating a memory-enhancing effect. GAL, utilized as a positive reference medication, elicits memory-enhancing effects, as seen by behavioral parameters in comparison to the Sco group (*p* < 0.0001), corroborating the findings from the Y-maze test. In the NOR test concerning AgNPs, one-way ANOVA indicated a substantial treatment effect on preference percentages [F (4, 45) = 64.39, *p* < 0.0001] (Figure 10). Zebrafish administered Sco exhibited a marked reduction in preference percentages (*p* < 0.0001) relative to the control group, whereas the administration of AgNPs enhanced preferences (*p* < 0.00001) for the novel object (N), indicating a memory-enhancing effect. GAL, utilized as a positive reference medication, induces memory-enhancing effects, as seen by behavioral characteristics in comparison to the Sco group (*p* < 0.0001), corroborating the findings from the Y-maze test.

Scopolamine is known to induce cognitive and behavioral impairments primarily through the blockade of muscarinic acetylcholine receptors, resulting in the disruption of cholinergic neurotransmission, elevated oxidative stress, mitochondrial dysfunction and the activation of neuroinflammatory cascades. These processes collectively contribute to neuronal damage and cognitive decline. In the present study, treatment with green-synthesized AgNPs markedly reversed scopolamine-induced deficits, suggesting that their neuroprotective effects may be mediated through multiple interrelated mechanisms involving antioxidant defense, cholinergic modulation and anti-inflammatory activity.

One plausible mechanism underlying this neuroprotection is the enhancement of the antioxidant system. AgNPs have been widely reported to elevate the activities of endogenous antioxidant enzymes such as superoxide dismutase, catalase and glutathione peroxidase while concurrently restoring glutathione levels and reducing lipid peroxidation markers such as malondialdehyde [25,26,27,28]. By scavenging ROS and stabilizing antioxidant enzyme conformations, AgNPs can re-establish redox homeostasis and protect neuronal membranes from peroxidative injury.

Green-synthesized AgNPs may also modulate cholinergic signaling, which is central to learning and memory. Several studies have shown that plant derived AgNPs, including those synthesized using *Heliotropium eichwaldi* [29] and *Mentha piperita* [30], exhibit significant acetylcholinesterase inhibitory activity in brain tissues, leading to increased synaptic acetylcholine levels and improved cognitive performance. In our study, the behavioral recovery and biochemical restoration observed after AgNP treatment could therefore be partially attributed to a similar mechanism, wherein acetylcholinesterase inhibition restored cholinergic transmission disrupted by scopolamine. This mechanism aligns with the therapeutic strategy of clinically used acetylcholinesterase inhibitors for memory impairment and Alzheimer’s-like models.

The anti-inflammatory properties of green-synthesized AgNPs also likely contribute to their neuroprotective efficacy. Phytochemicals from the plant extract used during nanoparticle synthesis act as capping and stabilizing agents, often rich in polyphenols and flavonoids that suppress the activation of pro-inflammatory signaling pathways. Previous reports demonstrated that AgNPs can downregulate cytokines such as tumor necrosis factor-alpha (TNF-α), interleukin-1 beta (IL-1β) and interleukin-6 (IL-6), primarily through the modulation of the NF-κB signaling cascade [31,32]. Such inhibition of neuroinflammatory mediators may prevent glial activation and secondary oxidative injury, preserving neuronal integrity in the hippocampal and cortical regions affected by scopolamine.

Accumulating evidence suggests that AgNPs may exert a stabilizing effect on mitochondrial integrity and apoptotic signaling. By restoring mitochondrial membrane potential and reducing the Bax/Bcl-2 ratio, AgNPs can limit the initiation of caspase-dependent apoptosis, thereby enhancing neuronal survival under oxidative or inflammatory stress [33]. These effects support the multifactorial neuroprotection observed in our scopolamine model, where both biochemical and behavioral outcomes improved following AgNP administration.

These findings support a comprehensive mechanism in which green-synthesized AgNPs mitigate scopolamine-induced neurodegeneration through synergistic actions on oxidative stress, cholinergic dysfunction and inflammation. The nanoparticles appear to restore redox balance, inhibit acetylcholinesterase and suppress pro-inflammatory cytokine production, ultimately preserving neuronal structure and function. This integrated mechanism is consistent with previous studies demonstrating that biogenic AgNPs possess potent antioxidant and anti-inflammatory properties that can counteract chemically induced neurotoxicity.

## 3. Materials and Methods

### 3.1. Plant Extract and Synthesis of AgNPs

The plant extract was obtained by direct collaboration with the Vanelli company, which is the single importer of *Lespedeza capitata* dried extract (4:1) in our country.

**Figure 11:**
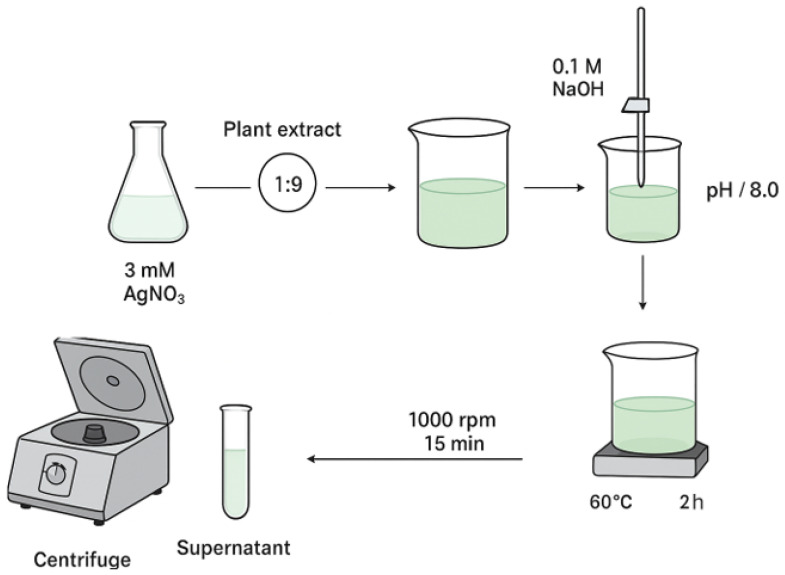
Graphical illustration of the synthesis and separation of AgNPs.

Synthesis was performed following a modified literature method [34], using aqueous AgNO_3_ at one concentration (3 mM) to ascertain the proper conditions optimal for AgNPs formation (Figure 11). Plant extract and AgNO_3_ solution were combined at a constant extract-to-salt volume ratio (1:9), maintained throughout all tests. The pH of the reaction was brought up to 8.0 with the gradual addition of 0.1 M NaOH while maintaining continuous stirring. The mixture was kept at 60 °C for 2 h with continuous mixing. After completion, the dispersion was left to cool to ambient temperature and subsequently centrifuged at 1000 rpm for 15 min. The resulting supernatant was collected for future tests confirming the formation of AgNPs.

### 3.2. Characterization of AgNPs

The characterization of AgNPs was conducted using spectroscopic (UV-Vis) and microscopic (Scanning Transmission Electron Microscopy-STEM) approaches, together with dynamic light scattering (DLS) and zeta potential assessments. The production of AgNPs was confirmed using UV-Vis spectroscopy, attributed to the surface plasmon resonance phenomenon, which reflects the collective oscillations of conduction electrons in metallic nanoparticles when exposed to light. The spectra were obtained with a Specord 210 Plus spectrophotometer (Analytik Jena, Thuringia, Germany) throughout the 200–800 nm range. The colloidal dispersion underwent DLS analysis with a Delsa Nano Submicron Particle Size Analyzer (Beckman Coulter Inc., Fullerton, CA, USA), yielding results on the hydrodynamic diameter and polydispersity index. The identical apparatus was employed to ascertain the zeta potential, which indicates the surface charge and colloidal stability of the produced AgNPs. The morphological characteristics and particle size distribution were examined via STEM utilizing a Hitachi High-Tech HT 7700 microscope (Hitachi High-Technologies Corporation, Tokyo, Japan), which provided micrographs for direct observation of the AgNPs.

### 3.3. Antioxidant Tests

#### 3.3.1. Ferrous Ion Chelation Assay

Fe^2+^ forms with ferrozine a pink complex that has a maximum absorption at 562 nm. The presence of a chelation complex reduces the formation of this complex.

In 0.2 mL of the sample, 0.74 mL acetate buffer 0.1 M (pH5.25), 0.02 mL ferrous sulphate 2 mM dissolved in hydrochloric acid 0.2 M were added; after stirring for 10–15 s, 0.04 mL of ferrocine 5 mM was added. After resting for 10 min in a dark place, the absorbance was tested at 562 nm, comparing it to a blank prepared in the same conditions (ferrous sulphate replaced with distilled water). A control solution and its blank were prepared from 0.2 mL double distilled water, 0.74 mL acetate buffer 0.1 M (pH 5.25) and 0.02 mL ferrous sulphate solution 2 mM in hydrochloric acid 0.2 M; after stirring for 10–15 s, 0.04 mL ferrocine 5 mM was added.

All the measurements were in sets of three, the results being represented as their mean ± the standard deviation.

#### 3.3.2. Hydroxyl Ion Scavenging Assay

The hydroxyl ion formed between the reaction of ferrous ions and hydrogen peroxide will add itself to salicylic acid, forming a pink–violet compound with a maximum absorbance at 562 nm.

In 0.225 mL of the sample, 0.750 mL 1.5 M iron (II) sulphate, 0.9 mL 20 mM sodium salicylate and 0.525 mL 6 mM hydrogen peroxide were added. The mixture was kept for 30 min at 37 °C, and after letting it cool to room temperature, the absorbance was registered for the sample at 562 nm and blank (the iron sulphate was replaced with water).

All the measurements were in sets of three, the results being represented as their mean ± the standard deviation.

#### 3.3.3. Lypoxigenase Inhibition

Natural compounds like flavanones and synthetic nanostructures containing silver can block 15-lipoxygenase, which leads to decreased linoleic acid oxidation and the reduction in the absorbance to 234 nm.

0.05 mL 15-lipoxygenase in borate buffer pH 9 was added to 0.05 mL of the sample and left to rest for 10 min at room temperature, after which 2 mL linoleic acid 0.16 mM in borate buffer 0.1 M pH 9 was added. The absorbance of this solution was registered at 234 nm, in the time interval of 0 to 120 s. The blank was prepared as well, replacing the sample with double distilled water.

All the measurements were in sets of three, the results being represented as their mean ± the standard deviation.

### 3.4. Animal Studies

All experimental procedures were performed using minimally invasive techniques and strictly adhered to humane endpoint guidelines to ensure the welfare of the animals. The study protocol was reviewed and approved by the Ethics Committee for Animal Research of Alexandru Ioan Cuza University of Iasi, Faculty of Biology (Iasi, Romania), under license no. BIO-UAIC-2023-1714, dated 6 July 2023. The experimental design complied with Directive 2010/63/EU of the European Parliament and of the Council of 22 September 2010 on the protection of animals used for scientific purposes, as well as with national regulations (approval no. Reg. 70/19/06/2023). At the beginning of the study, 100 adult wild-type short-fin zebrafish (*Danio rerio*) of both sexes (1:1 male-to-female ratio; 3–4 months old; 3–4 cm in length) were obtained from the European Zebrafish Resource Center at the Institute of Toxicology and Genetics, Germany. The fish were acclimatized for at least 14 days in the experimental facility before testing. They were maintained in groups of 10 per 24 L thermostated tanks (26 ± 1 °C) equipped with water filtration and aeration systems (dissolved oxygen: 7.20 mg/L) using Tetratec^®^ air pumps (Tetra, Melle, Germany) [35,36]. Animals were housed under a 14:10 h light/dark photoperiod and fed twice daily with Norwin Norvitall flake food (Norwin, Gadstrup, Denmark). Additionally, the sample size determination (*n* = 10 zebrafish per group) was conducted using InVivoStat and validated through an R-based statistical power analysis to ensure adequate experimental sensitivity [37]. Using a significance threshold of 0.05, the statistical power for detecting a biologically meaningful difference of 20% was calculated to be 93%. The data analysis, reporting and experimental design were performed in accordance with the ARRIVE guidelines to ensure methodological rigor and reproducibility [38] for planning and organizing animal testing and research, respectively. All statistical analyses were performed in a blinded manner to prevent experimenter bias and ensure objective data interpretation. Following acclimation, zebrafish were randomly assigned to experimental groups: control, scopolamine (Sco, 100 µM) or treatment with silver nanoparticles (AgNPs, 6 µg/L). Galantamine (GAL, 1 mg/L), a cholinesterase inhibitor, served as the reference compound in the Y-maze test, while imipramine (IMP, 20 µg/L), a tricyclic antidepressant, was used as the positive control in the novel tank diving test. The doses of AgNPs and Sco were selected based on previous studies [39,40]. AgNPs were diluted in 1% DMSO and administered by immersion for 1 h daily for 8 consecutive days, while Sco (100 µM) was administered 30 min before each behavioral test. The control group was exposed to unchlorinated water containing 1% DMSO only.

Throughout the experimental period, the health and welfare of all animals were closely monitored. No procedures induced severe pain or lasting distress, and no mortality occurred during housing or behavioral testing.

#### 3.4.1. Y-Maze Test Assay

The response to novelty in zebrafish was assessed using the Y-maze task [41]. The location in the Y-maze task was considered to be a memory index [42]. The apparatus consisted of a Y-maze glass tank with three identical arms (25 cm long, 8 cm wide and 15 cm high), filled with 3 L of the home aquarium water. The water in the Y-maze was 13 cm. Explicit geometric shapes (squares, circles and triangles) were placed on the outer walls and were visible from the inside. The Y-maze test consisted of two trials separated by a 1 h interval. During the first trial, 1 h after AgNPs LCE treatment, the fish could freely swim in the start arm and the other arm for 5 min while the novel arm was blocked by a dividing wall. In the second trial, the wall was removed, and the fish could explore for 5 min all three arms, including the novel environment constituted by the novel arm. Fish were placed in different arms as starting points, and the maze was rotated in each experiment to randomize the maze cues. The water was changed between groups and trials. The behavior was fully analyzed using the ANY-Maze^®^ software v7.49 (Stoelting CO, Wood Dale, IL, USA), assessing time spent in the novel arm (% of total arm time), total distance traveled (m) and turn angle (°).

#### 3.4.2. Novel Tank Diving Test (NTT) Method

The zebrafish swimming behavior within the in vivo tasks was recorded with a Logitech C922 Pro HD Stream webcam (Logitech, Lausanne, Switzerland), and the recordings were analyzed using ANY-maze software v7.49 (Stoelting Co., Wood Dale, IL, USA).

In the NTT, the zebrafish exhibit robust behavioral responses to novelty-provoked anxiety. The NTT protocol applied in this study was described before by Rehman et al. (2010) [43] and Omidvari et al. (2011) [44]. The testing apparatus consisted of a trapezoidal glass tank filled with 1.5 L of home tank water, with the following dimensions: 23.9 cm along the bottom × 28.9 cm at the top × 15.1 cm high with 15.9 cm along the diagonal side, 7.4 cm wide at the top and 6.1 cm wide at the bottom. The fish were individually placed in the testing tank, and their behavior was recorded for 6 min with a webcam placed 40 cm in front of the tank. The tank was virtually divided into the top zone and the bottom zone, respectively. To measure anxiety-like behavior and the locomotor activity of the zebrafish, we used the behavioral endpoints described previously by Rehman et al. (2010) [43].

#### 3.4.3. Novel Object Recognition Test (NOR) Method

The zebrafish swimming behavior within the in vivo tasks was recorded with a Logitech C922 Pro HD Stream webcam (Logitech, Lausanne, Switzerland), and the recordings were analyzed using ANY-maze software v7.49 (Stoelting Co., Wood Dale, IL, USA).

The NOR is a commonly used behavioral assay for the investigation of memory performance in zebrafish [45]. The experimental apparatus consists of a 20 L glass tank (30 × 30 × 30 cm) filled with 6 cm of water. Before training, each animal was habituated to the apparatus in the absence of the objects for 5 min twice a day (5 h interval between habituation sessions) over 3 consecutive days. On the 4th day, in the training phase, the animals were exposed to two identical red cubes for 10 min. After the training phase, the animals were submitted to a retention interval of 1 h. In the test phase, a novel object (N, yellow cube) replaced one of the copies of the familiar objects (F, blue cube), and the exploration time of each object was evaluated for 10 min. The preference percentages were the behavioral parameters evaluated in this test. The preference percentages were calculated as [time of exploration of NO/time of exploration of FO + time of exploration of NO × 100].

#### 3.4.4. Statistical Analysis

All given results represent the mean values of three determinations ± the standard deviation. Also, for the integration of the obtained results, and to establish the statistical significance, one-way ANOVA and Tukey’s post hoc analyses were conducted. All given data with *p* < 0.01 were considered statistically significant, and those with *p* < 0.00001 were very significant.

## 4. Limitations of the Study

Despite the positive results obtained, certain limitations must be acknowledged. The production and characterization of AgNPs were performed on a limited sample set, potentially neglecting variations in particle morphology, size distribution or surface chemistry between batches. The discrepancies identified between the hydrodynamic diameter obtained from DLS and the particle size determined from STEM imaging underscore the fundamental methodological distinctions between these techniques. Although these discrepancies were elucidated by solvation and surface capping effects, supplementary analyses such as TEM, FTIR or XPS could have provided greater insight into the precise surface composition and chemical state of the nanoparticles.

Another limitation concerns the assessment of the stability of the AgNPs. The reduced zeta potential indicates moderate electrostatic stability; however, the long-term colloidal stability has not been evaluated in various environmental or physiological conditions. Since biogenic nanoparticles may undergo oxidation, aggregation or surface modification over time, future studies should include assessments of extended storage and stability under biological conditions.

With regard to the antioxidant assays, although statistically significant differences were observed between the crude plant extract and the manufactured nanoparticles, the experimental design was confined to in vitro chemical models of antioxidant capacity. These assays do not accurately represent the complex redox environment found in living organisms. The extrapolation of the antioxidant capacity of AgNPs to in vivo systems necessitates careful consideration. Moreover, the precise roles of distinct phytochemicals acting as capping agents in the exhibited antioxidant effects remain inadequately characterized.

The behavioral experiments performed on zebrafish exhibit a restricted sample size for each experimental group, which may limit the statistical power and generalizability of the findings. Zebrafish are an important vertebrate model for neurobehavioral research, but differences in metabolism, blood–brain barrier permeability and nanoparticle biodistribution between species may limit the direct applicability of these results to higher vertebrates or humans. Moreover, while the behavioral evaluations (Y-maze, NTT and NOR) revealed significant cognitive and anxiolytic-like effects of both the plant extract and AgNPs, no biochemical or histopathological correlates were investigated to validate these functional findings at the molecular level.

## 5. Conclusions

This study demonstrates the successful green-synthesis of AgNPs using plant extract, yielding distinct colloidal systems with different physicochemical properties. UV-Vis analysis confirmed the formation of AgNPs, presenting a broader peak around 450 nm, consistent with more polydisperse structures.

The synthesis procedure successfully generated AgNPs with desirable nanoscale proportions. Characterization revealed a hydrodynamic diameter of 90.3 nm and a polydispersity index of 0.3366, while STEM images confirmed predominantly spherical particles with sizes ranging from 15 to 20 nm. Although the zeta potential values indicated reduced electrostatic stability compared to flaxseed-derived AgNPs (−44 mV reported in the literature), the nanoparticles maintained acceptable colloidal behavior.

In vivo assays using zebrafish demonstrated significant biological activity of the AgNPs against scopolamine-induced deficits. In the Y-Maze test, AgNPs at µg/L significantly reversed amnesic effects (*p* = 0.001) and increased locomotion, with the total distance traveled and turn angle both improved (*p* < 0.00001). In the NTT, AgNPs increased time spent in the top zone (*p* < 0.00001), decreased freezing duration (*p* < 0.00001) and counteracted the hypolocomotor effect induced by Sco (*p* < 0.001 for 3 µg/L; *p* < 0.00001 for 6 µg/L). In the NOR test, AgNPs significantly improved novel object preference percentages (*p* < 0.00001), restoring recognition memory impairments caused by scopolamine.

Collectively, these findings demonstrate that the synthesized AgNPs possess stable physicochemical properties and exert robust neuroprotective, anxiolytic and memory-enhancing effects in zebrafish. Their consistent performance across multiple behavioral tests and effective doses (3–6 µg/L) underscores their potential as promising candidates for biomedical applications in the management of neurocognitive disorders.

This study demonstrates that silver nanoparticles synthesized using *Lespedeza capitata* extract offer distinct advantages in terms of biological activity, particularly antioxidant and anti-inflammatory effects. Nanoformulation not only compensates for the poor bioavailability of lipophilic compounds but also significantly enhances their efficacy compared to the crude extract. Importantly, no signs of toxicity were observed in the experimental models, supporting the safety of the obtained nanoparticles. Taken together, these findings suggest that plant-mediated silver nanoparticles hold promise for the prevention and management of conditions driven by oxidative stress and chronic inflammation, including those with a neurodegenerative component.

## Figures and Tables

**Table 1:** The ferrous chelation potential of AgNPs and plant extract.

AgNPs	4.140625 μg/mL	8.28125 μg/mL	16.5625 μg/mL	33.125 μg/mL	66.25 μg/mL	132.5 μg/mL	265 μg/mL
	2.89 ± 0.26	6.64 ± 0.85	12.10 ± 0.74	20.42 ± 0.59	29.40 ± 0.81	41.46 ± 0.96	63.18 ± 0.91
Plant extract	0.1625 mg/mL	0.3125 mg/mL	0.625 mg/mL	1.25 mg/mL	2.5 mg/mL	5 mg/mL	10 mg/mL
	1.38 ± 0.07	4.27 ± 0.21	12.24 ± 0.11	19.14 ± 0.07	32.63 ± 0.46	36.82 ± 0.32	40.37 ± 1.27
*p*-value	0.021	0.013	0.876 (ns)	0.072 (ns)	0.004	0.006	<0.001

*p* < 0.05; *p* < 0.01; *p* < 0.001; *p* ≥ 0.05 = ns (not significant).

**Table 2:** The hydroxyl ion scavenging activity.

AgNPs	4.140625 μg/mL	8.28125 μg/mL	16.5625 μg/mL	33.125 μg/mL	66.25 μg/mL	132.5 μg/mL	265 μg/mL
	1.20 ± 0.01	2.66 ± 0.08	6.46 ± 0.02	14.24 ± 0.06	27.70 ± 0.07	40.96 ± 0.04	56.25 ± 0.21
Plant extract	0.1625 mg/mL	0.3125 mg/mL	0.625 mg/mL	1.25 mg/mL	2.5 mg/mL	5 mg/mL	10 mg/mL
	4.99 ± 0.05	7.42 ± 0.03	9.15 ± 0.07	12.62 ± 0.02	20.15 ± 0.07	34.43 ± 0.12	53.79 ± 003
*p*-value	<0.001	<0.001	0.002	0.014	<0.001	0.009	0.038

**Table 3:** The lipoxygenase inhibition activity of the AgNPs and plant extract.

AgNPs	4.140625 μg/mL	8.28125 μg/mL	16.5625 μg/mL	33.125 μg/mL	66.25 μg/mL	132.5 μg/mL	265 μg/mL
	0.92 ± 0.18	3.23 ± 0.34	7.07 ± 0.25	14.67 ± 0.37	17.68 ± 0.47	31.14 ± 0.95	41.36 ± 0.61
Plant extract	0.1625 mg/mL	0.3125 mg/mL	0.625 mg/mL	1.25 mg/mL	2.5 mg/mL	5 mg/mL	10 mg/mL
	1.84 ± 0.09	3.75 ± 0.44	11.37 ± 0.66	19.37 ± 0.99	32.35 ± 1.34	53.31 ± 1.25	67.35 ± 0.52
*p*-value	0.031	0.212 (ns)	0.006	0.015	<0.001	<0.001	<0.001

## Data Availability

All of the newly obtained data is available in this article.
